# An unusual cause of adrenal insufficiency with elevation of 17-hydroxyprogesterone: case report

**DOI:** 10.1186/s12902-023-01374-7

**Published:** 2023-05-29

**Authors:** Claudia Teti, Giampaolo Bezante, Federico Gatto, Keyvan Khorrami Chokami, Manuela Albertelli, Marco Falchi, Giulio Bovio, Sandro Teresio Nati, Diego Ferone, Mara Boschetti

**Affiliations:** 1 Endocrinology Unit, ASL 1, Imperia, Italy; 2 Cardiology Unit, IRCCS Ospedale Policlinico San Martino, Genoa, Italy; 3 Endocrinology Unit, IRCCS Ospedale Policlinico San Martino, Genoa, Italy; 4 Endocrinology Unit, Department of Internal Medicine & Medical Specialties (DiMI), IRCCS Ospedale Policlinico San Martino, University of Genoa, Genoa, Italy; 5 Radiology Unit, Biomedical, Genoa, Italy; 6 Interventional Radiology Unit, IRCCS Ospedale Policlinico San Martino, Genoa, Italy; 7 Hematology Unit, Montallegro Clinic, Genoa, Italy

**Keywords:** Adrenal incidentaloma, Addison disease, Lymphoma, Corticosteroids, Case report

## Abstract

**Background:**

We present an intriguing case of primary adrenal lymphoma, with associated primary adrenal insufficiency (PAI), in a patient presenting a transitory partial 21-hydroxylase deficiency during the active phase of the adrenal disease.

**Case presentation:**

An 85-years old woman was referred because of worsening asthenia, lumbar pain, generalized myalgia and arthralgia. During investigations a computed tomography (CT) scan evidenced two large bilateral adrenal masses, highly suspicious for primary adrenal tumor. The hormonal assessment revealed very low levels of morning plasma cortisol and 24-h urinary cortisol, elevated ACTH levels with low plasma concentration of aldosterone, pointing to the diagnosis of PAI. After diagnosis of PAI our patient started glucocorticoid and mineralcorticoid replacement therapy with clinical benefit. In order to further characterize the adrenal lesions, adrenal biopsy, was performed. The histology revealed a high grade non-Hodgkin lymphoma with an immunophenotype consistent with intermediate aspects between diffuse large B-cell and Burkitt lymphoma, with a high proliferation index (KI-67 > 90%). The patient received chemotherapy with epirubicin, vincristine, cyclophosphamide, and rituximab, associated with methylprednisolone that resulted in a complete clinical and radiological remission within one year. After 2 years from the diagnosis and a total of 6 cycles of rituximab, the patient was in good clinical condition and was taking only the replacement therapy for PAI. The patient initially presented also a slight increase of 17-hydroxyprogesterone (17-OHP) for age that normalize after resolution of lymphoproliferative disease.

**Conclusions:**

In the presence of bilateral adrenal disease and/or in the presence of signs and symptoms of PAI clinicians must exclude the presence of PAL. The evidence of elevated ACTH-stimulated 17-OHP levels also in patients with other adrenal masses, together with the detection of elevated basal 17-OHP levels in our patient make it more plausible, in our view, an effect of the lesion on the “healthy” adrenal tissue residue than a direct secretory activity by the adrenal tumor.

## Background

Primary adrenal insufficiency (PAI) is a critical life-threatening condition due to the incapacity of the adrenal cortex to produce sufficient amounts of glucocorticoids and/or mineralocorticoids, first described by Thomas Addison [1]. The etiology of PAI should be rapidly determined in all patients in order to optimize the primary and etiological treatments [2]. Majority of PAI are caused by autoimmune or infectious diseases, other causes include adrenal masses or injuries, congenital adrenal hyperplasia or hypoplasia, ACTH insensitivity, iatrogenic causes [2]. Adrenal incidentalomas are highly prevalent and, therefore, frequently detected by imaging studies unrelated to the suspect of adrenal diseases [3].

Primary adrenal lymphoma (PAL) is a rare incidentaloma, and adrenal insufficiency as clinical presentation is highly variable, from 11 to 61% in literature [4–6], and occurs more commonly in cases with bilateral extensive invasion [7–9]. PAL is an extremely rare but rapidly progressive disease and it generally carries a poor prognosis [10].

PAL is suspect on the basis of clinical and imaging assessment, however should be definitively confirmed by histopathology. Recently it has been proposed [11] a quick and efficient diagnostic method to guide PAL detection.

We present an intriguing case of PAL, with associated PAI, in a patient presenting a transitory partial 21-hydroxylase deficiency during the active phase of the adrenal disease.

## Case presentation

An 85-years old woman was referred because of worsening asthenia, lumbar pain, generalized myalgia and arthralgia. The patient had a history of toxic multinodular goiter, hypertension, atrial fibrillation treated with metimazole, irbesartan and dabigratan, respectively. An accurate cardiological evaluation, including electrocardiogram and echocardiography, excluded that the impairment of the symptoms was due to the cardiovascular alterations. Moreover, general hematologic tests, as well as the thyroid function resulted normal.

**Fig. 1:**
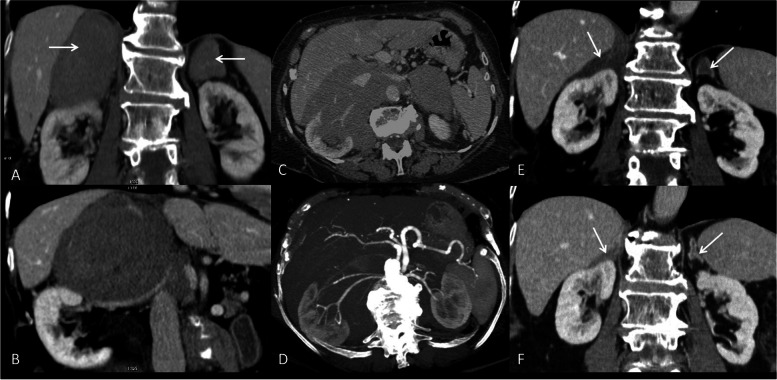
First CT scan showing **A** asymmetric enlargement of the right adrenal mass imprinting the liver and displacing caudally the normal gland; **B** detail of the right adrenal mass that displaces the surrounding structures. The kidney is displaced caudally and renal vessels are marked but without signs of infiltration; **C** A second CT scan revealing the dimensional increase of the adrenal masses. The right one incorporates all the surrounding vascular structures up to the left vascular bundle; **D** Maximum Intesity Projection (MIP) reconstruction shows that the right vessels near the adrenal mass are stretched but without signs of infiltration; **E** Three months after therapy CT scan revealed a complete remission of the right mass. The adrenal gland shows normal morphology with densification of the surrounding fatty tissue. The left adrenal mass is greatly reduced in size; **F** Eighteen months after diagnosis CT scan showed a complete remission of both the adrenal masses

Clinical picture was negative, except for NYHA class II, whereas chest x-ray showed the presence of a very small nodule in the middle lobe of the right lung. However, a computed tomography (CT) scan did not confirm the x-ray picture, whereas evidenced two large bilateral adrenal masses (85 × 75x62 mm on the right, and 62 × 59x24 mm on the left side), with positive contrast enhancement and reduced washout on delayed images. The masses displayed a right extension to the aorto-caval space, incorporating the renal vessels and imprinting the inferior vena cava (Fig. 1A, B). These radiological features were highly suspicious for primary adrenal tumor. Normal values of the urinary metanephrines ruled out the hypothesis of pheochromocytoma. Moreover, the diagnosis of adrenal carcinoma was unlikely, since circulating levels of androgens (testosterone, Dehydroepiandrosterone-sulfate, Delta4 androstenedione) were low or normal, except for an increase of 17-hydroxyprogesterone (17-OHP). This finding was confirmed through two repeated measurements (2.88 mcg/L and 2.79; normal value between 0.13 and 0.51). The 17-OHP levels were assayed by radioimmunoassay. Assay sensitivity was 0.01 ng/dL, whereas intra- and inter-assay coefficients of variation were both 9.0%). The remaining hormonal assessment revealed very low levels of morning plasma cortisol and the 24-h urinary free cortisol in the lower range of normality, elevated ACTH levels with low plasma concentration of aldosterone, pointing to the diagnosis of PAI (Table 1).

**Table 1:** Hormonal assessment at diagnosis

Assay	Value	Normal range
S-Morning cortisol	3.0	3.7–19.4 mcg/dl
24-h urinary free cortisol	10.75	4.30–176.00 mcg/24 h
S-ACTH	213.0	0.0–46.0 pg/ml
S-Aldosterone	15.2	25.2–392.0 ng/L
S-Dehydroepiandrosterone-sulfate	150	350–4300 mcg/L
S-Delta4 androstenedione	0.9	0.3–3.3 mcg/L
S-17-hydroxyprogesterone	2.88	0.13–0.51 mcg/L
S-Testosterone	15.1	0.0–62.0 ng/dl
24-h urinary metanephrines	17.64	50.00–300.00 mcg/24 h
24-h urinary normetanephrines	210.10	90.00–400.00 mcg/24 h

Radiological assessment ruled out the presence of adrenal haemorrhages that may occur during chronic anticoagulant therapy, particularly during dabigratan treatment. Although previously described [12], this is a very rare, difficult to diagnose and potentially fatal condition if not recognized and promptly treated.

**Fig. 2:**
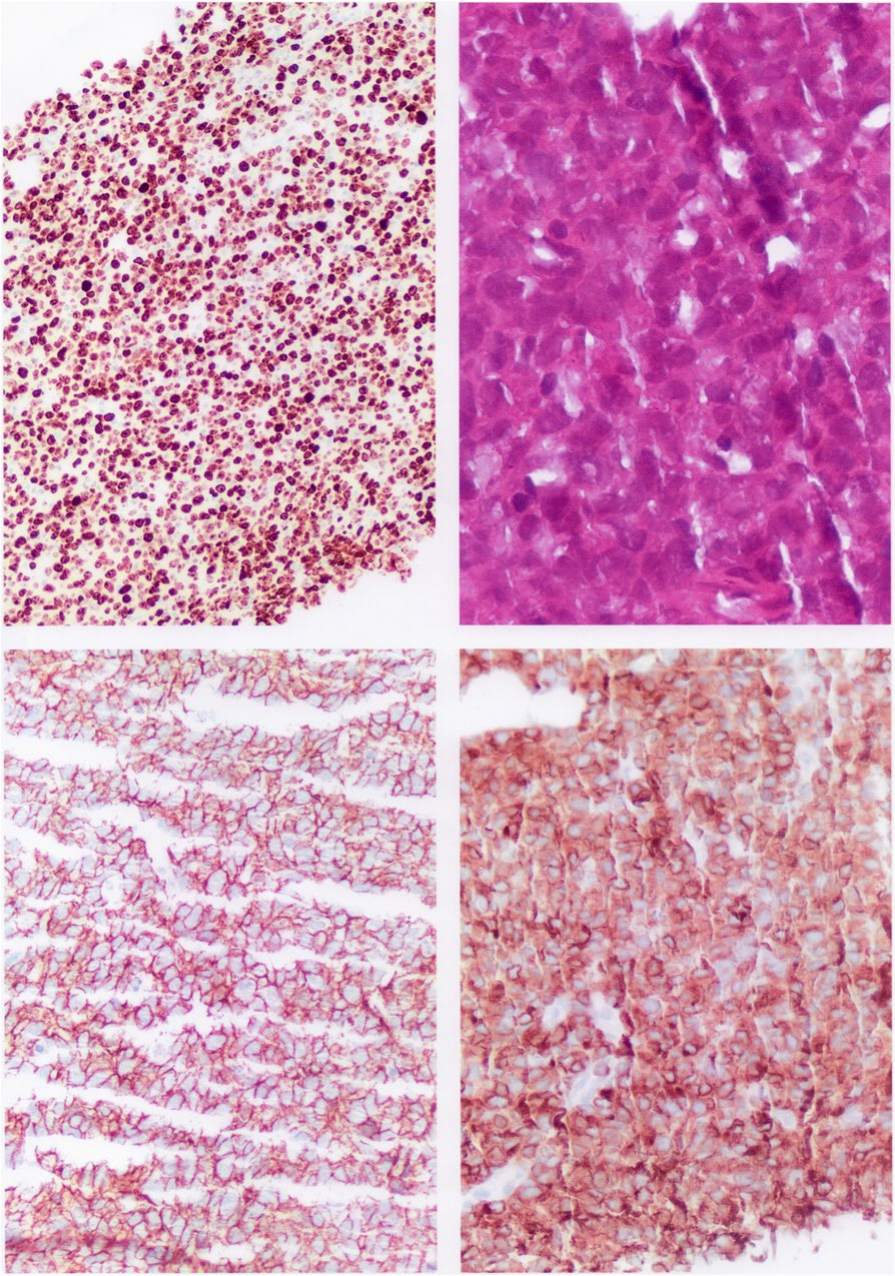
Histology and immunohistochemistry. **A** High-power wiew hematoxylin–eosin stained section showing a diffuse proliferation of medium to large cells with scant cytoplasm and hyperchromatic nucleus; **B** High percentage of the tumor cells showing nuclear immunoreactivity for MIB-1; **C**, **D** Positive CD79 and CD20 immunoreactivity of the tumor cells

After diagnosis of PAI our patient started glucocorticoid and mineralcorticoid replacement therapy (cortisone acetate and fludrocortisone) with a partial clinical benefit. In order to further characterize the adrenal lesions, adrenal biopsy, initially refused by the patient, was performed 4 months later, when a second CT scan depicted a significant increase of the two adrenal masses (the right resulted 150 × 110x120 and the left 110 × 95x44 mm) (Fig. 1C, D). The histology revealed a high grade non-Hodgkin lymphoma with an immunophenotype consistent with intermediate aspects between diffuse large B-cell and Burkitt lymphoma, with a high proliferation index (KI-67 > 90%) (Fig. 2).

Due to the complex clinical presentation, as well as the atypical classification, the patient received chemotherapy with epirubicin, vincristine, cyclophosphamide, and rituximab (anti CD20 monoclonal antibody), associated with methylprednisolone that resulted in a complete clinical and radiological remission within one year (Fig. 1E, F). After 2 years from the diagnosis and a total of 6 cycles of rituximab, the patient is in very good clinical condition and is currently taking only the replacement therapy for the adrenal insufficiency.

Considering the high levels of 17-OHP at diagnosis we hypothesized a partial 21-hydroxylase deficiency, that could have led, together with the mass effect, to the onset of PAI. However, after two years ACTH test evidenced a normal 21-hydroxylase activity (baseline and stimulated 17-OHP were 0.34 and 0.46 mcg/L, respectively).

## Discussion and conclusions

Although lymphoma may occasionally involve the adrenal glands as part of a generalized disease process, PAL is rare, accounting for about 3% of extranodal presentation [13, 14]. Indeed, among endocrine organs, thyroid is the most commonly gland involved in lymphoproliferative disorders [15]. PAL is usually bilateral, while secondary adrenal gland involvement is typically unilateral [6, 16].

The radiological characteristics of the adrenal masses in our patient were highly indicative of neoplastic disease, with Hounsfield units higher than 10, slower washout in delayed contrast-enhanced CT, size and rapid growth in short time [17]. However, the adrenal biopsy confirmed the malignancy, and was crucial for the identification of PAL. In fact, on CT and MR imaging, PAL usually appear as a heterogeneous mass, sometimes with cystic aspects due to tumor necrosis, rarely it appears as homogeneous neoplasm that, conversely, is typical of secondary adrenal involvement by lymphoma [4].

Clinical presentation of PAL is variable and includes B-symptoms, pain, fatigue, anorexia, gastrointestinal and neurological symptoms [5]. Symptoms of adrenal insufficiency such as hypotension, vomiting, fatigue and skin hyperpigmentation occur especially if there is extensive bilateral adrenal involvement [18–20]. Nevertheless, PAI in PAL can be clinically relevant even in cases with only minimal local involvement of the adrenal glands [21].

Corticotropin stimulation test is the gold standard for diagnosis of PAI. However, in patients with confirmed cortisol deficiency accompanied by plasma ACTH two times above the upper limit of normality, as well as typical clinical signs and symptoms of PAI, as in our patient, the baseline evaluation is sufficient for the diagnosis [2].

As is known, urinary free cortisol is not a gold standard test for diagnosis and a normal value does not exclude the presence of PAI [2].

Interestingly, in our patient, at diagnosis, 17-OHP levels were high for age and sex but this result was not confirmed after two years when ACTH test evidenced a normal 21-hydroxylase activity. Therefore, a transient 21-hydroxylase deficiency status occurred during the active phase of the disease. This phenomenon is very interesting and could explain the lymphoproliferative disease occurrence of PAI also in the adrenal lymphomatous diseases of smaller dimension, in which the mass effect does not justify the presence of PAI.

Patients with non-functioning adrenal adenomas have often an increased plasma 17-OHP during ACTH stimulation [22–27]. In a retrospective study (about 160 patients with adrenal masses), basal plasma 17-OHP was normal in patients with all types of tumors (non-functioning, cortisol- and aldosterone-producing adenomas, adrenal cysts, pheochromocytomas). Whereas, ACTH-stimulated plasma 17-OHP was abnormally increased in 53% and 31% of patients with non-functioning and aldosterone-producing adenomas, respectively. After unilateral adrenalectomy, this hormonal abnormality disappeared in most, although not all patients with adrenal tumors [28]. Different authors evidenced the presence of correlation between ACTH-stimulated plasma 17-OHP concentrations and the size of non-functioning adrenal adenomas before adrenal surgery [25, 28, 29], assuming a possible role of the mass itself in the 17-OHP secretion. Other studies revealed in non-functioning adrenocortical adenoma P450c21 mRNA levels and activity of P450c21cytochrome comparable with normal adrenal tissue, suggesting that these are not the only determinant of the activity of the 21-hydroxylase enzyme and tissue 17-OHP content [30, 31].

The evidence of elevated ACTH-stimulated 17-OHP levels also in patients with other adrenal masses, apart from non-functioning adenoma (aldosterone-producing adenomas; adrenal cysts; pheochromocytomas) [21], together with the detection of elevated basal 17-OHP levels in our patient (lymphoproliferative disease) make it more plausible, in our view, an effect of the lesion on the “healthy” adrenal tissue residue than a direct secretory activity by the adrenal tumor.

In the presence of bilateral adrenal disease with instrumental characteristics described above and/or in the presence of signs and symptoms of PAI should be excluded the presence of PAL [32]. In the comprehensive study of adrenal function in adrenal lymphomas, in selected cases, it could be useful to assess a complete corticosteroid profile including precursors together with 17-OHP levels.

## Data Availability

All the data generated and/or analyzed during this study are included in this published article.

## Abbreviations

PAI: Primary Adrenal Insufficiency
CT: Computerized Tomography
ACTH: Adrenocorticotropic hormone
17-OHP: 17-Hydroxyprogesterone
PAL: Primary Adrenal Lymphoma
MR: Magnetic Resonance
